# Supplementary material for: Epigenomic diagnosis and prognosis of Acute Myeloid Leukemia
Source: Nat Commun. 2025 Jul 29;16:6961. doi: 10.1038/s41467-025-62005-4 (PMC12307604; doi:10.1038/s41467-025-62005-4)
Supplement: Supplementary file 1 — Supplementary Information [file 41467_2025_62005_MOESM1_ESM.pdf]

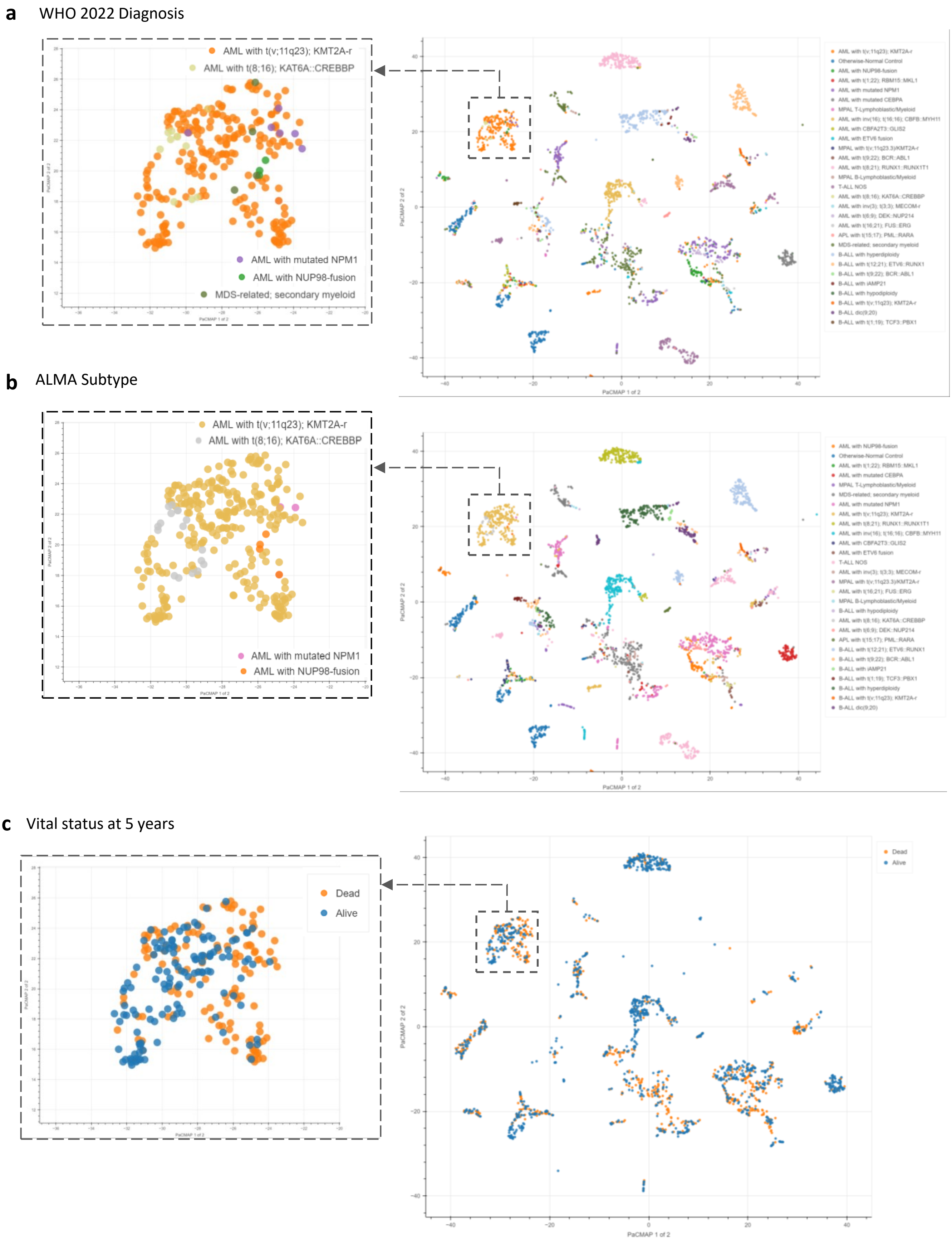

Supplementary Fig. 1: Case study for AML with t(v;11q23); KMT2A-rearrangement cluster.
a WHO 2022 Diagnosis **b** ALMA Subtype and **c** Vital status.

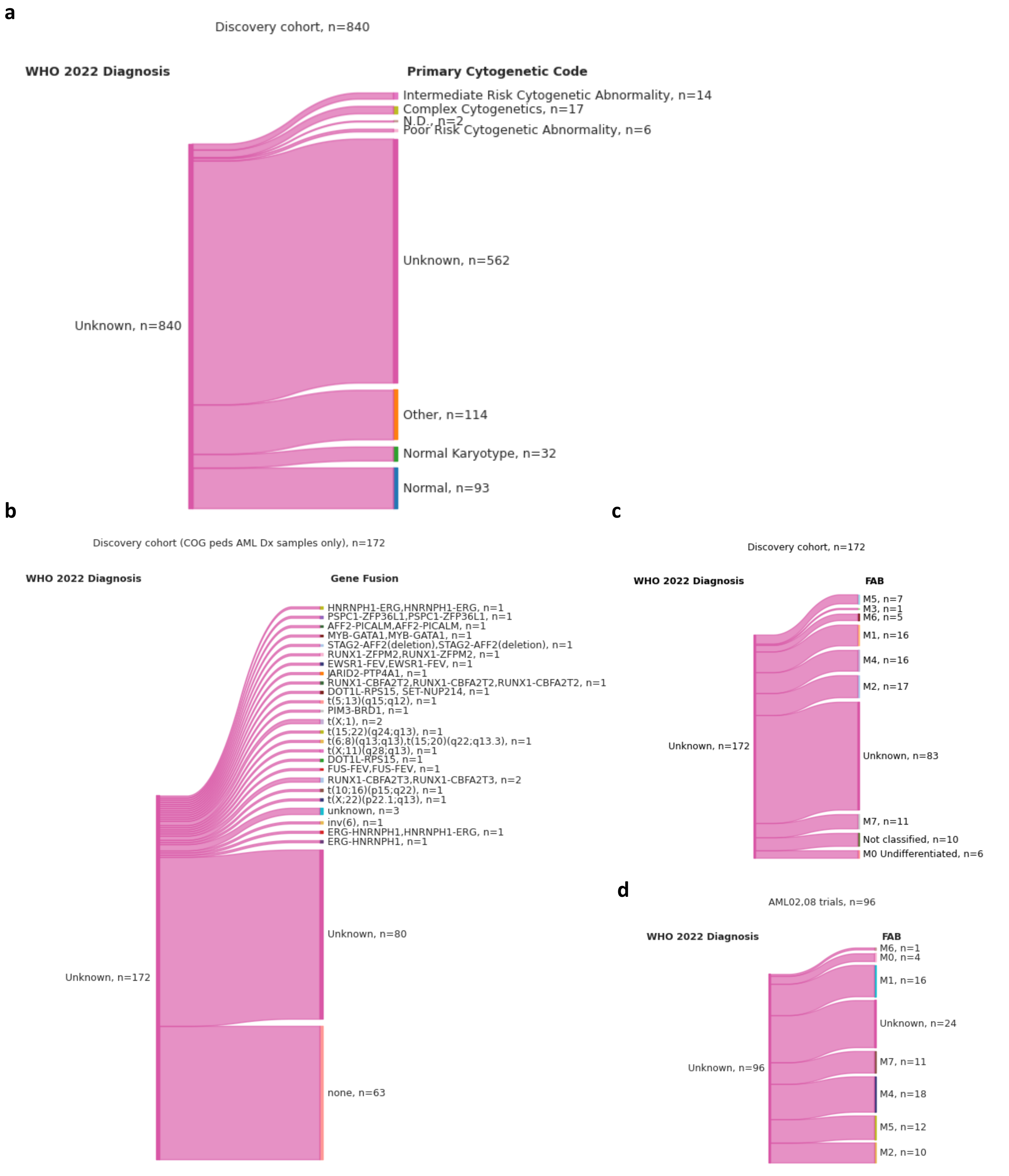

Supplementary Fig. 2: Reason for unclassified samples in WHO 2022 diagnosis. **a** WHO 2022 diagnosis versus primary cytogenetic code available through datasets from all cohorts. **b** WHO 2022 diagnosis versus gene fusions in COG datasets. Fusions listed here did not unambiguously fall into diagnostic categories specified by WHO 2022. WHO 2022 diagnosis versus FAB in **c** discovery and **d** AML02,08 test cohort. FAB, French-American-British classification of AML. Individual n numbers are indicated in the figures.

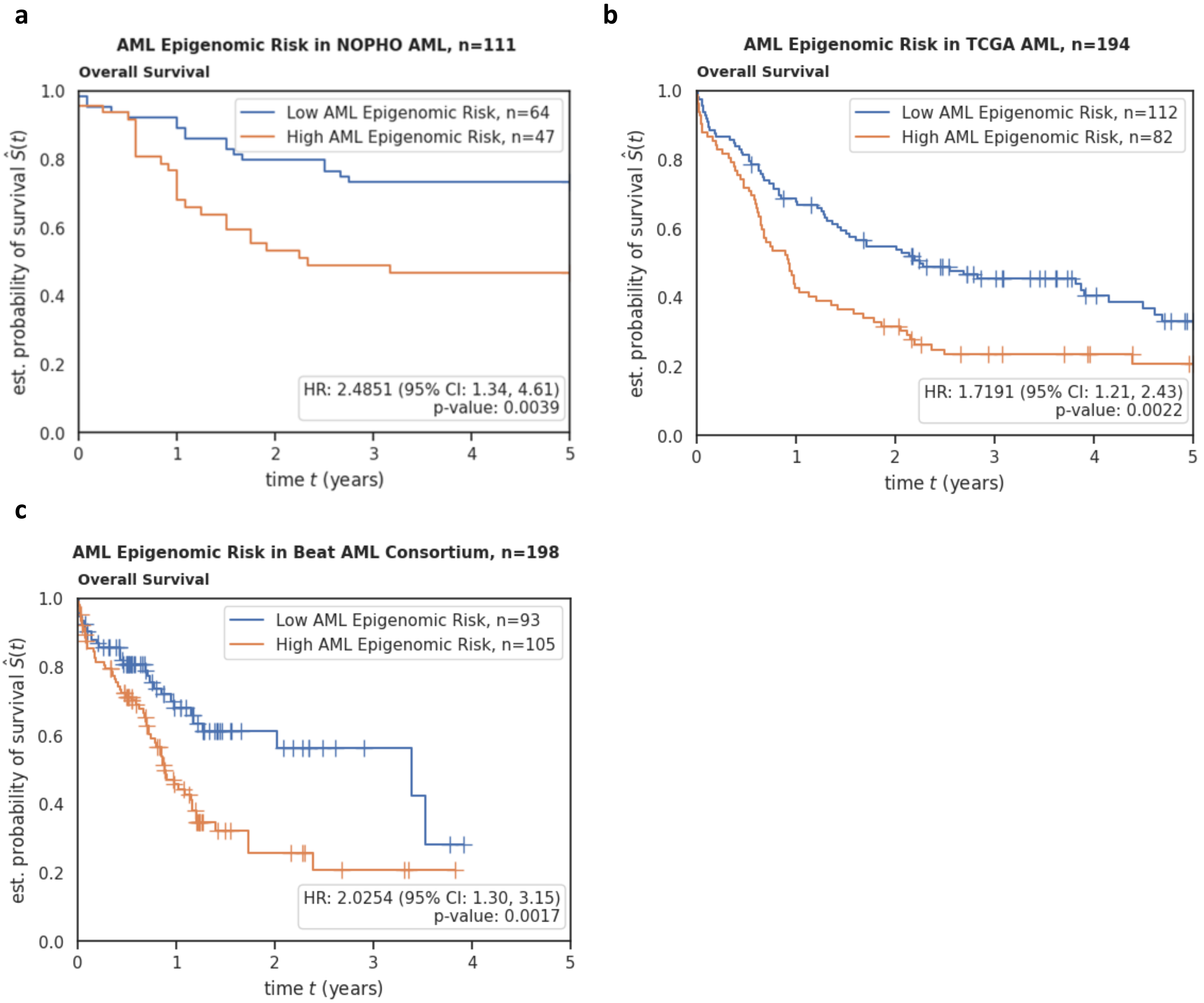

Supplementary Fig. 3: OS outcomes by AML Epigenomic Risk in NOPHO AML, TCGA, and Beat AML cohorts. a NOPHO AML (n=111). **b** TCGA (n=194). **c** Beat AML Consortium (n=198). Hazard ratios derive from Cox PH regression with two-sided hypothesis tests.

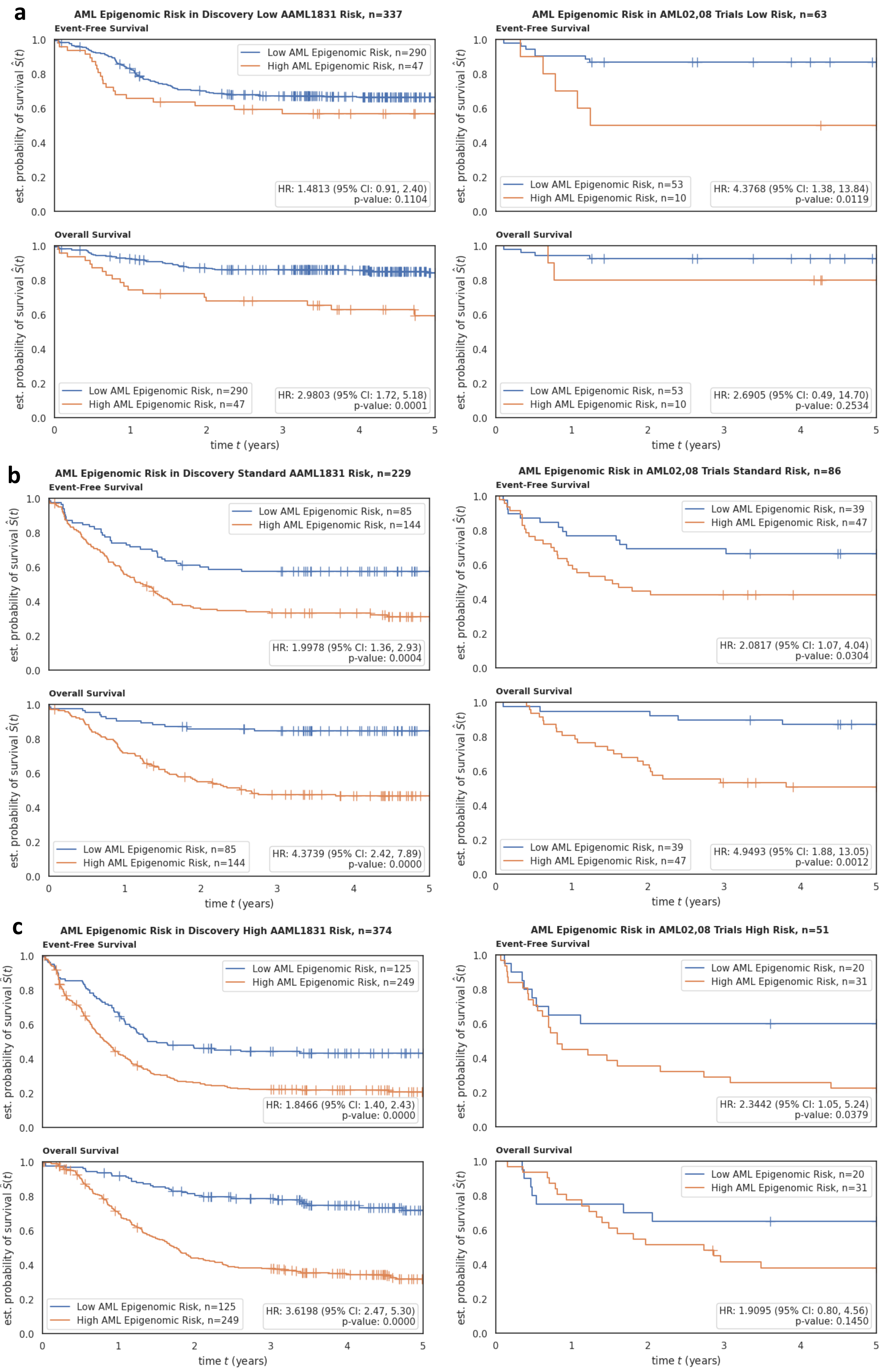

Supplementary Fig. 4: EFS and OS outcomes by AML Epigenomic Risk divided by risk groups. **a** Low risk. **b** Standard risk. **c** High risk. Hazard ratios derive from Cox PH regression with two-sided hypothesis tests.

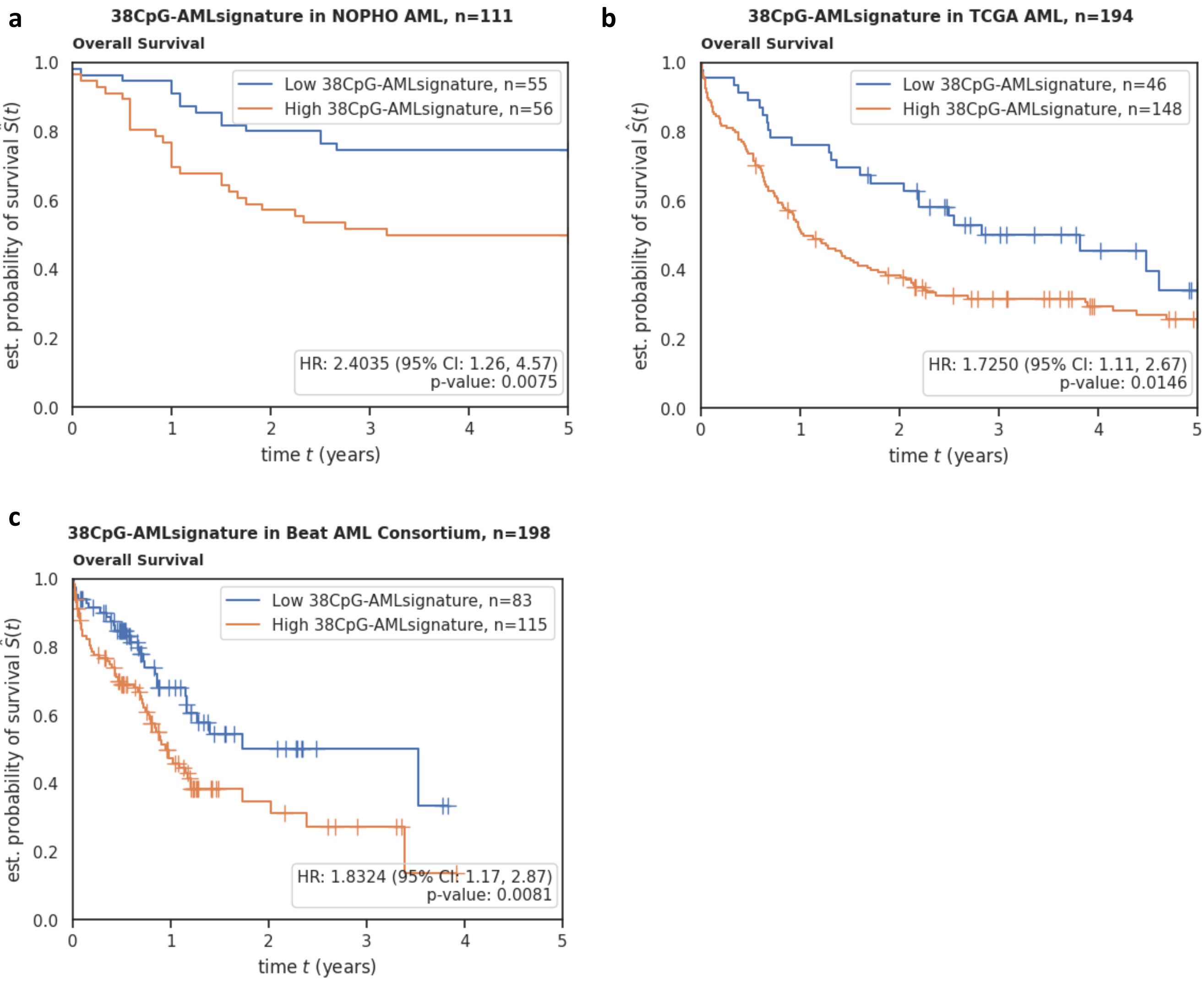

Supplementary Fig. 5: OS outcomes by 38-CpG AML Signature in NOPHO AML, TCGA, and Beat AML cohorts.

a NOPHO AML (n=111). **b** TCGA (n=194). **c** Beat AML Consortium (n=198). Hazard ratios derive from Cox PH regression with two-sided hypothesis tests.

a

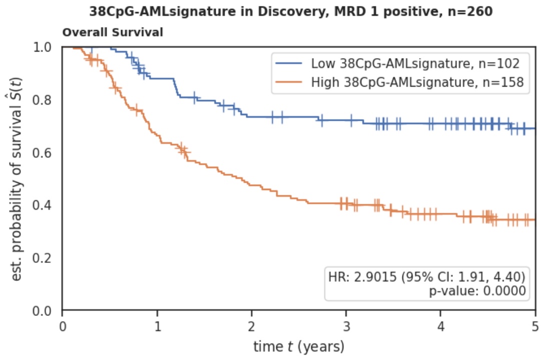

	0	1	2	3	4	5
**Low 38CpG-AMLsignature, n=102**						
At risk	102	85	68	64	53	0
Censored	0	5	8	11	21	73
Events	0	12	26	27	28	29
**High 38CpG-AMLsignature, n=158**						
At risk	158	100	70	57	38	0
Censored	0	6	8	11	25	61
Events	0	52	80	90	95	97

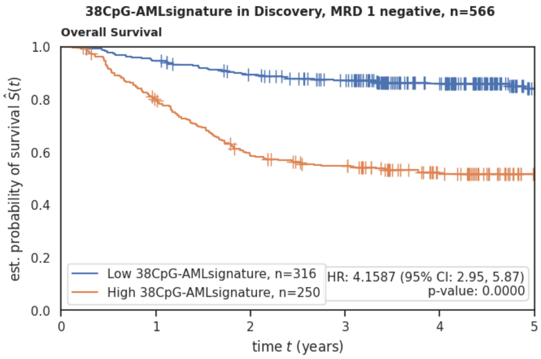

	0	1	2	3	4	5
**Low 38CpG-AMLsignature, n=316**						
At risk	316	298	275	251	180	0
Censored	0	1	8	25	93	271
Events	0	17	33	40	43	45
**High 38CpG-AMLsignature, n=250**						
At risk	250	197	141	126	90	0
Censored	0	4	7	13	43	133
Events	0	49	102	111	117	117

b

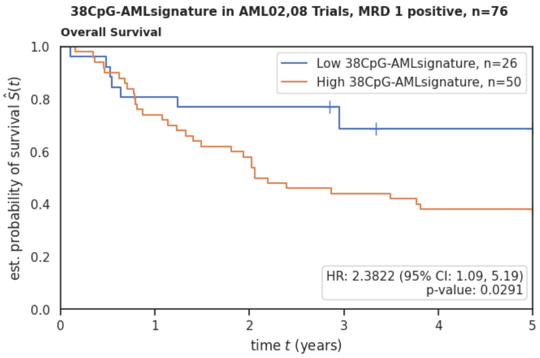

	0	1	2	3	4	5
**Low 38CpG-AMLsignature, n=26**						
At risk	26	21	20	17	16	0
Censored	0	0	0	1	2	18
Events	0	5	6	8	8	8
**High 38CpG-AMLsignature, n=50**						
At risk	50	37	29	22	19	0
Censored	0	0	0	0	0	19
Events	0	13	21	28	31	31

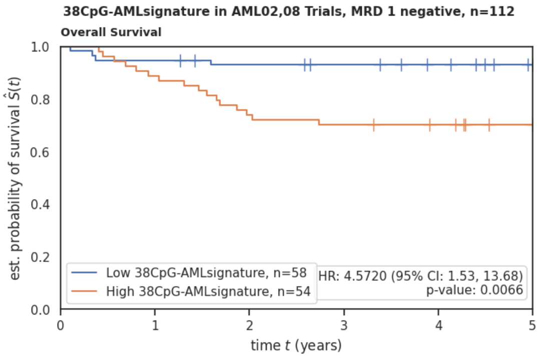

	0	1	2	3	4	5
**Low 38CpG-AMLsignature, n=58**						
At risk	58	55	52	50	47	0
Censored	0	0	2	4	7	54
Events	0	3	4	4	4	4
**High 38CpG-AMLsignature, n=54**						
At risk	54	48	40	38	36	0
Censored	0	0	0	0	2	38
Events	0	6	14	16	16	16

Supplementary Fig 6: Patient outcomes by *38-CpG AML Signature groups* within MRD1 positive and MRD1 negative groups

OS within MRD1 positive and within MRD1 negative groups by ***38-CpG AML Signature groups*** *in* **a)** discovery cohort and **b)** AML02,08 test cohort. Hazard ratios derive from Cox PH regression with two-sided hypothesis tests.

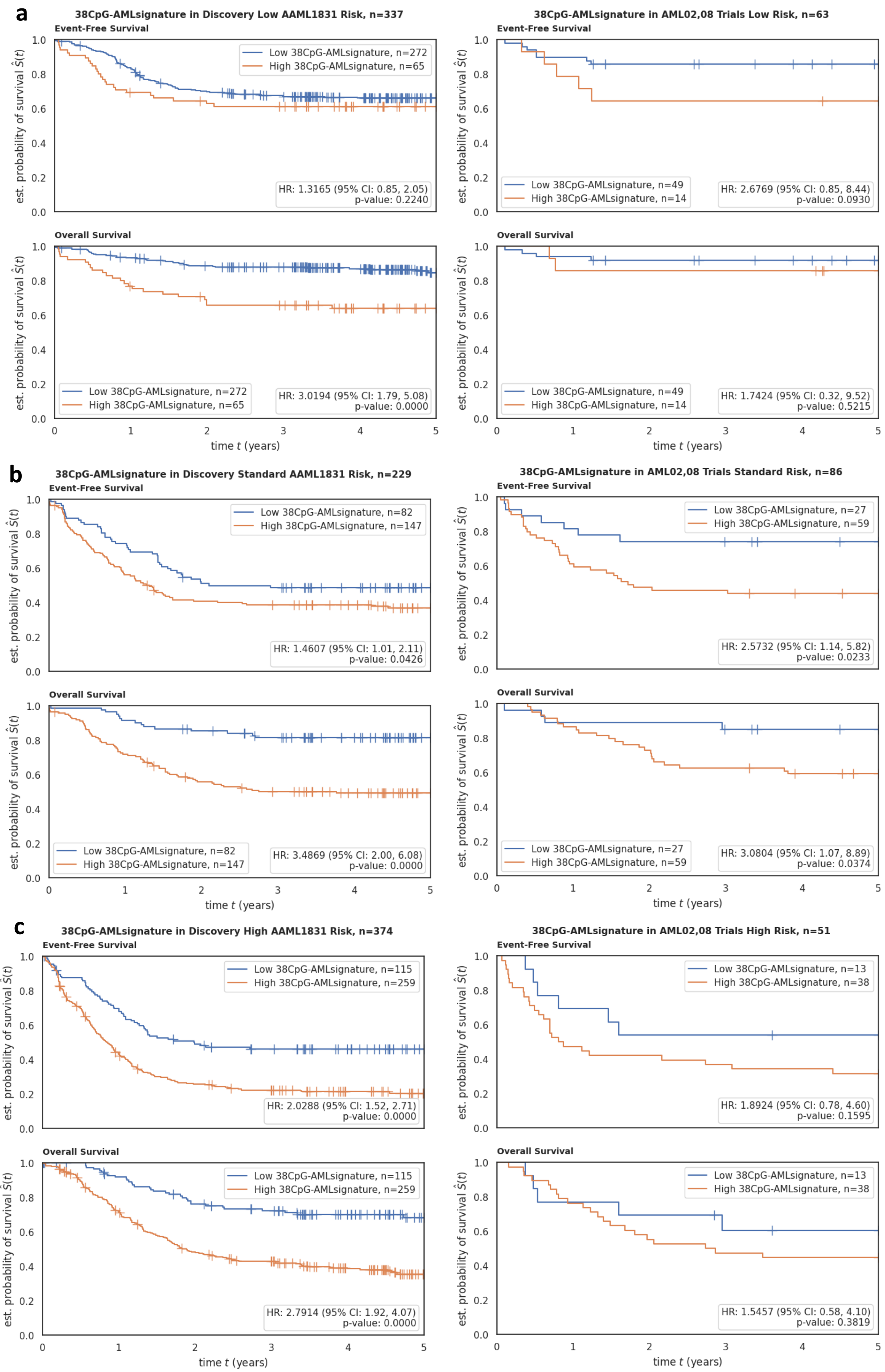

Supplementary Fig. 7: EFS and OS outcomes for 38CpG AML signature by risk group in discovery and AML02,08 test cohorts.
a) Low risk. **b)** Standard risk. **c)** High risk. Hazard ratios derive from Cox PH regression with two-sided hypothesis tests.

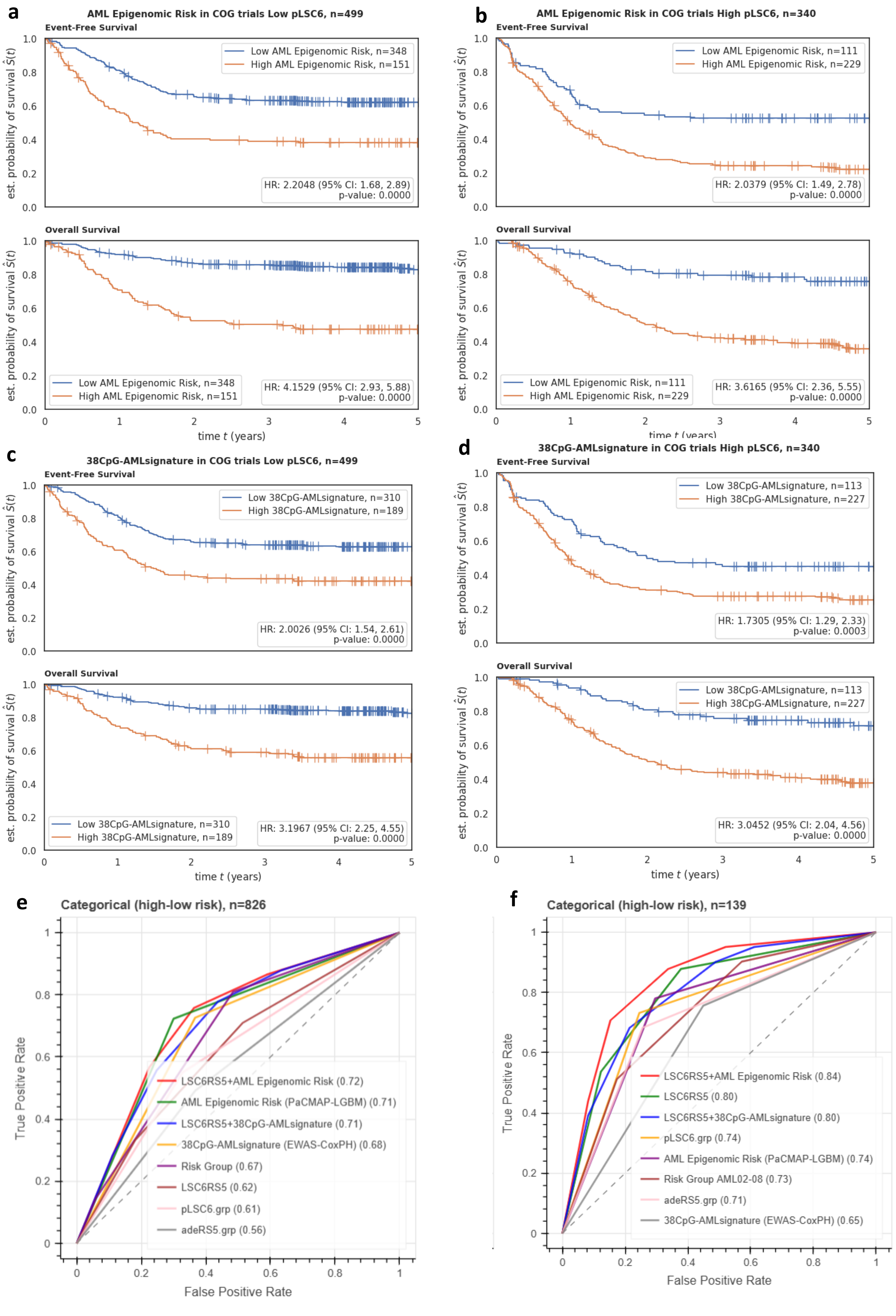

Fig. continued →

g

Variable	Observed C - index	BootStrap Median Cindex	BootStrap lower95CI	BootStrap upper95CI
Risk Group	0.633	0.633	0.605	0.661
pLSC6	0.59	0.589	0.555	0.625
LSC6RS5 (4 group)	0.586	0.589	0.552	0.616
38CpG- AML Epigenomics Score	0.719	0.719	0.689	0.749
AML Epigenomic Risk	0.713	0.713	0.684	0.742
Risk Group + pLSC6	0.639	0.641	0.606	0.668
Risk Group+LSC6RS5	0.645	0.648	0.61	0.672
Risk Group+30 CpG Epigenomics Score	0.724	0.725	0.693	0.754
Risk Group + AML Epigenome Risk	0.717	0.718	0.686	0.745
Risk Group + pLSC6+ 38 CpG Epigenomics Score	0.725	0.726	0.693	0.754
Risk Group + pLSC6+ AML Epigenome Risk	0.717	0.719	0.686	0.745
Risk Group +LSC6RS5+38 CpG Epigenomic Score	0.728	0.73	0.696	0.756
Risk Group+LSC6RS5+AML Epigenome Risk	0.717	0.72	0.686	0.744

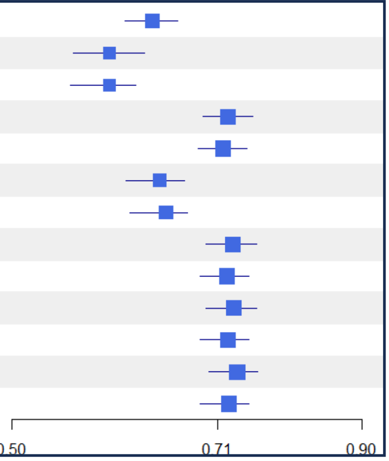

* All models were stratified by clinical trial treatment arm

h

Variable	Observed C - index	BootStrap Median Cindex	BootStrap lower95CI	BootStrap upper95CI
Risk Group	0.686	0.688	0.605	0.764
pLSC6	0.713	0.715	0.621	0.801
LSC6RS5 (4 group)	0.737	0.745	0.655	0.806
38CpG-Epigenomic score	0.636	0.639	0.54	0.73
AML Epigenomic Risk	0.692	0.695	0.6	0.782
Risk Group + pLSC6	0.725	0.732	0.629	0.809
Risk Group+LSC6RS5	0.768	0.778	0.68	0.84
Risk Group+38CpG_ Epigenomic Score	0.722	0.725	0.635	0.806
Risk Group + AML Epigenome Risk	0.729	0.739	0.639	0.796
Risk Group + pLSC6+ 38 CpG Epigenomic Score	0.755	0.763	0.661	0.838
Risk Group + pLSC6+ AML Epigenome Risk	0.738	0.754	0.639	0.806
Risk Group +LSC6RS5+38 CpG	0.783	0.794	0.693	0.853
Risk Group+LSC6RS5+AML Epigenome Risk	0.78	0.792	0.693	0.845

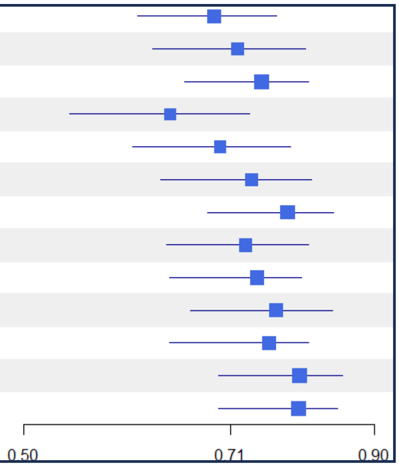

* All models were stratified by clinical trial treatment arm

Supplementary Fig. 8: Epigenomic models add additional predictive value to previously published transcriptomic and genomic scores.

Survival curves for EFS and OS in discovery cohort by *AML Epigenome Risk* within **a** low or **b** high pLSC6 score groups (as reported in [18]) and **c** and **d** *38-CpG AML Signature.* ROC curves showing the predictive value for diagnostic risk group for OS, current *AML Epigenome Risk* and *38-CpG AML Signatures, previously reported pLSC6, ADERS5 and integrated LSC6RS5 (as* defined by Elsayed et al [18,19]) as well as combination of DNA methylation and transcriptomic scores. Legend and AUC values are provided as inset for **e** discovery cohort and **f** AML02,8 test cohort. Bootstrap C-indices derived from Cox models for scores based on methylation and transcriptomic data, either individually or in various combinations with the risk group, evaluated across repeated bootstrap samples for **g** discovery and **h** AML02,8 test cohort. Hazard ratios derive from Cox PH regression with two-sided hypothesis tests.

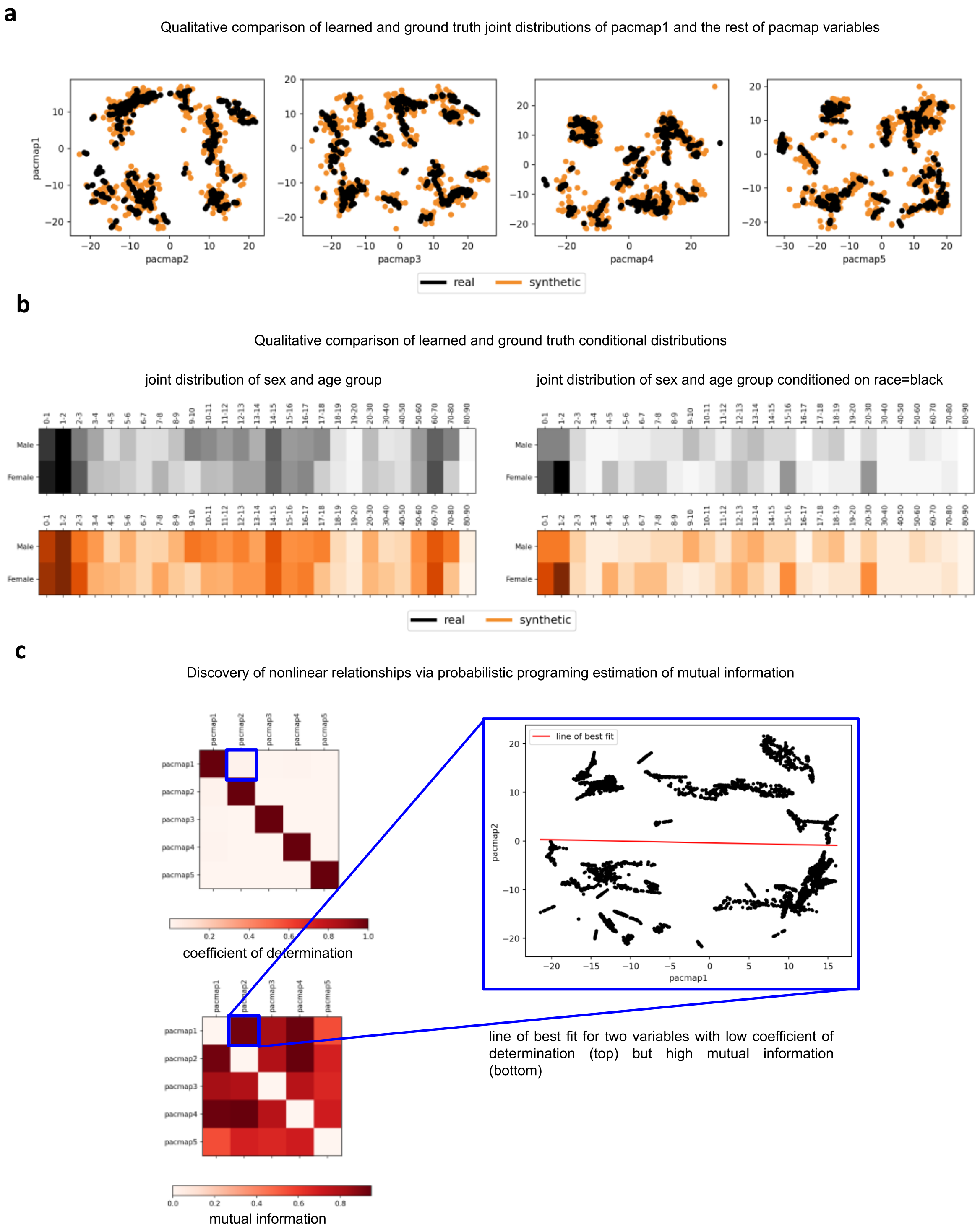

Supplementary Fig. 9: Overview of the learned generative population model.
a Two-dimensional scatter plots of ground truth PaCMAP variables overlaid on data generated from the learned population model. **b** Visualization of the joint distribution of sex and age (binned) before and after conditioning on race. Both before and after conditioning, the joint distribution of data generated from the model matches the empirical joint distributions. Subfigures A and B show that synthetic data from the generative model captures the statistical properties of the real data while at the same time diminishing privacy concerns. **c** Comparison of mutual information and coefficient of determination as measures of dependency among variables in the data. Linear statistics such as the coefficient of determination are not sensitive to non-linear dependencies in the data. Mutual information achieves higher sensitivity to non-linear dependencies without sacrificing specificity. Our probabilistic program representation allows for efficient and straightforward calculation of metrics such as mutual information.

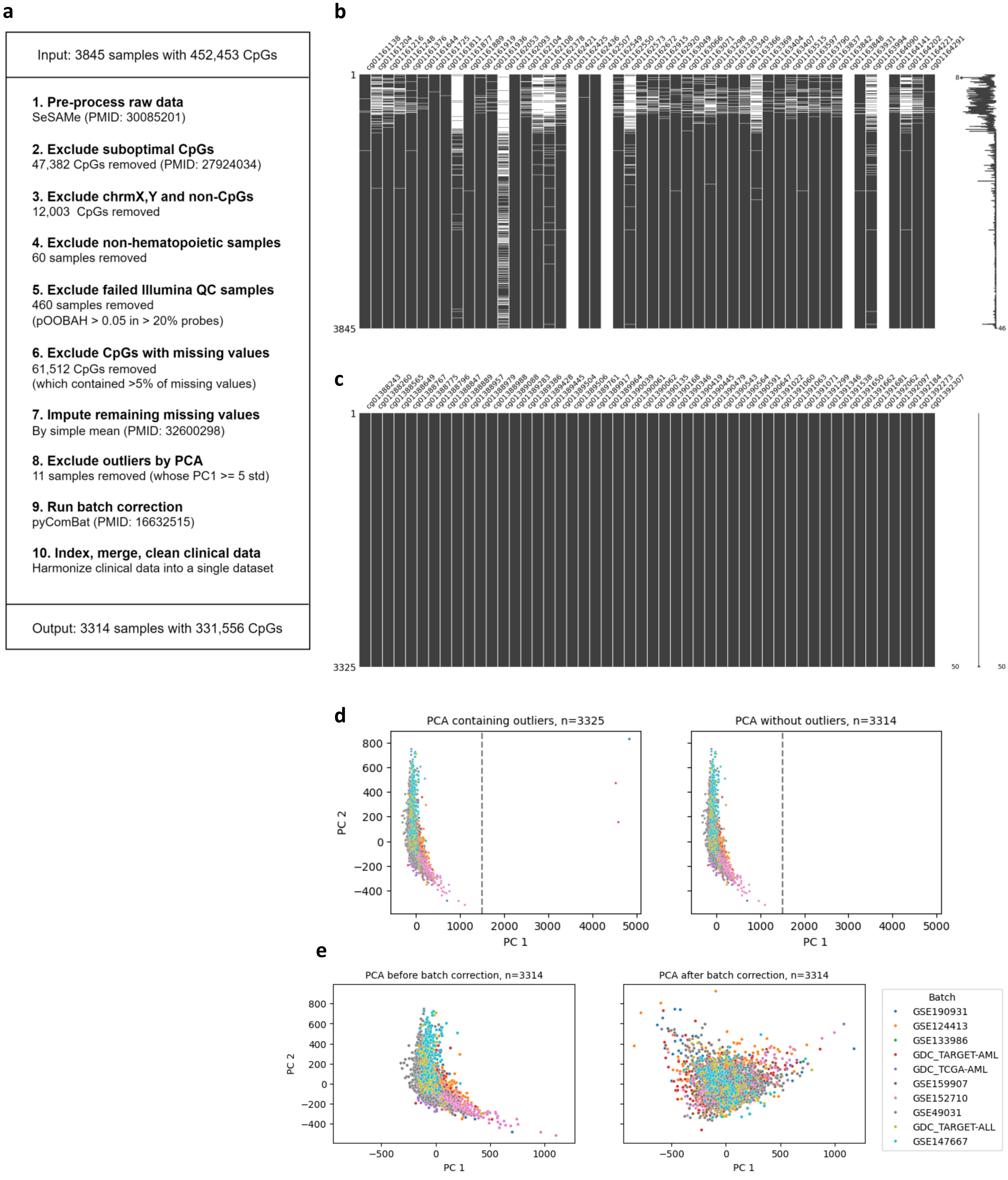

Supplementary Fig. 10: Pre-processing pipeline of methylation array cohorts.
a Overview of the preprocessing pipeline applied to the dataset, starting with 3,845 samples and 452,453 CpGs**. b** Visualization of missing data before and **c** after preprocessing. PCA plots illustrating the effects of **d** outlier removal and **e** batch correction. Color coding in PCA plots represents different dataset batches. Individual n numbers are indicated in the figures.

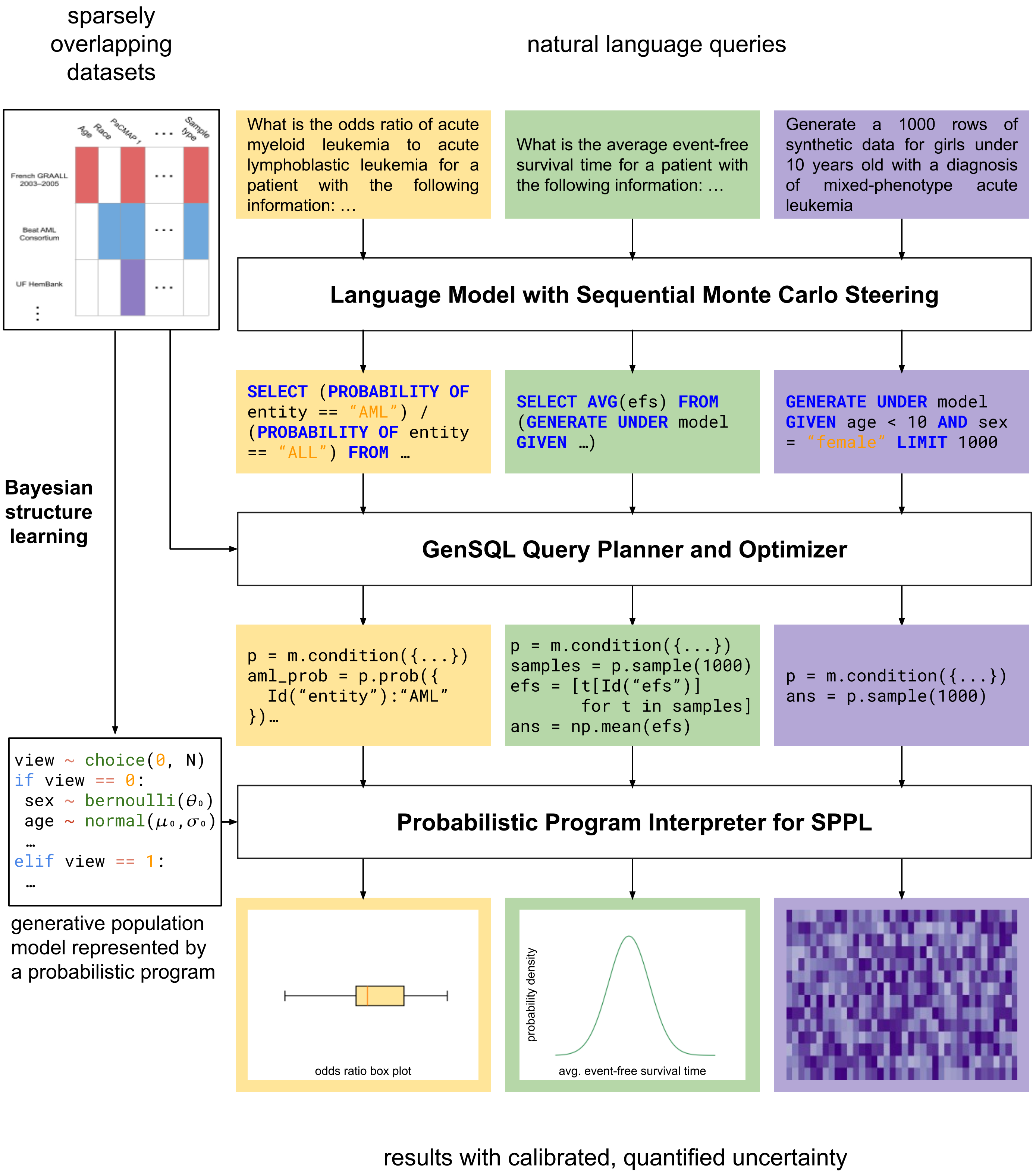

Supplementary Fig. 11: Overview of the workflow of generative population models (GPMs). We feed the ALMA dataset (16% sparse) to the Bayesian structure learning component of GenSQL to obtain a generative model of the full dataset represented by a probabilistic program in SPPL. Users can then supply natural language prompts to a steered large language model which outputs structured GenSQL queries. These queries are optimized and lowered to SPPL programs which are then executed against the model and the dataset to obtain results with calibrated quantified uncertainty. In summary, a GPM allows for interacting with the data through natural language by efficiently automating a significant proportion of the data science pipeline.

Supplementary Tables
Supplementary Table 1: Datasets and patient distribution in the ALMA discovery cohort
Supplementary Table 2: Patient characteristics for the three models in the discovery cohort.
Supplementary Table 3: Patient characteristics by AML Epigenomic Risk groups in the discovery and AML02,08 test cohorts.
Supplementary Table 4: 38 CpG AML signature coefficients and genomic loci according to hg38.
Supplementary Table 5: Patient characteristics by 38CpG AML signature groups in the discovery and AML02,08 test cohorts.
Supplementary Table 6: Specimen-to-result nanopore sequencing results.

Supplementary Table 1: Datasets and patient distribution in the ALMA discovery cohort

Dataset	Disease	Data Source	Initial Sample Size	Trial/study supplementary references	Trial/study PMIDs
COG_AAML1031*	Pediatric, AML	GSE190931	1048	[1,2]	36815378, 32029509
COG_AAML0531*	Pediatric, AML	GSE124413	500	[3,4]	34793513, 25092781
Japanese AML-05*	Pediatric, AML	GSE133986	64	[5,6]	35008106, 25975190
AML_TARGET#	Pediatric, AML	GDC_TARGET-AML	317	[4,7-9]	29227476, 25092781, 21766293, 18000167
AML_TCGA#	Adult AML	GDC_TCGA-AML	194	[10]	23634996
BeatAML*	Adult AML	GSE159907	316	[11,12]	33707228, 30333627
MDS_tAML#	Adult t-AML, MDS	GSE152710	166	[13]	33446256
Nordic_ALL#	Pediatric ALL	GSE49031	945	[14,15]	24063430, 20010622
ALL_TARGET*	Pediatric ALL	GDC-TARGET-ALL	102	[16]	30209392
Tcell_ALL_GRAAL*	Pediatric and Adult ALL	GSE147667	155	[17]	34039737

Note: Illumina450K array *Illumina EPIC array. Harmonized dataset available through ALMA v0.2.0[18].

Supplementary Table 2: Patient characteristics for the three models in the discovery cohort.

	Characteristics	ALMA Subtype	AML Epigenomic Risk & 38 CpG AML Signature
n		2471	946
Age (years), mean (SD)		19.2 (19.7)	9.4 (6.3)
Age group (years), n (%)	≥10	528 (47.4)	463 (48.9)
	<10	586 (52.6)	483 (51.1)
Sex, n (%)	Female	711 (50.5)	468 (49.5)
	Male	697 (49.5)	478 (50.5)
Race or ethnic group, n (%)	White	1064 (80.5)	697 (79.1)
	Black or African American	131 (9.9)	102 (11.6)
	Asian	65 (4.9)	43 (4.9)
	American Indian or Alaska Native	7 (0.5)	5 (0.6)
	Other	48 (3.6)	28 (3.2)
	Pacific Islander	7 (0.5)	6 (0.7)
Hispanic or Latino ethnic group, n (%)	Hispanic or Latino	209 (19.6)	185 (20.2)
	Not Hispanic or Latino	858 (80.4)	731 (79.8)
MRD 1 Status, n (%)	Positive	284 (29.6)	260 (31.5)
	Negative	675 (70.4)	566 (68.5)
Leucocyte counts (10^9/L), n (%)	≥30	579 (52.4)	467 (49.4)
	<30	526 (47.6)	479 (50.6)
BM leukemic blasts (%), mean (SD)		65.7 (24.1)	63.8 (24.5)
Risk Group, n (%)	High Risk	198 (14.2)	129 (13.8)
	Standard Risk	628 (45.0)	454 (48.7)
	Low Risk	570 (40.8)	349 (37.4)
FLT3 ITD, n (%)	Yes	180 (16.2)	165 (17.5)
	No	932 (83.8)	779 (82.5)
Clinical Trial, n (%)	AAML03P1	62 (2.5)	36 (3.8)
	AAML0531	517 (20.9)	507 (53.6)
	AAML1031	495 (20.0)	403 (42.6)
	BM normal AAML0531	41 (1.7)	
	Beat AML Consortium	192 (7.8)	
	CCG2961	31 (1.3)	
	CETLAM SMD-09 (MDS-tAML)	166 (6.7)	
	French GRAALL 2003–2005	141 (5.7)	
	Japanese AML05	9 (0.4)	
	NOPHO ALL92-2000	641 (25.9)	
	TARGET ALL	56 (2.3)	
	TCGA AML	120 (4.9)	

Supplementary Table 3: Patient characteristics by AML Epigenomic Risk groups in the discovery and AML02,08 test cohorts.

		AML Epigenomic Risk in discovery cohort, n=946			AML Epigenomic Risk in AML02,08 cohort, n=200		
		High	Low	P-Value	High	Low	P-Value
n		442	504		88	112	
Age (years), mean (SD)		8.7 (6.5)	10.0 (6.2)	**0.002***	7.9 (6.1)	9.4 (5.8)	0.083*
Age group (years), n (%)	≥10	200 (45.2)	263 (52.2)	**0.039**	35 (40.7)	60 (53.6)	0.098
	<10	242 (54.8)	241 (47.8)		51 (59.3)	52 (46.4)	
Sex, n (%)	Female	215 (48.6)	253 (50.2)	0.680	38 (43.2)	48 (42.9)	1.000
	Male	227 (51.4)	251 (49.8)		50 (56.8)	64 (57.1)	
Race or ethnic group, n (%)	White	323 (78.2)	374 (79.9)	0.971	63 (73.3)	79 (70.5)	0.688
	Black or African American	52 (12.6)	50 (10.7)		14 (16.3)	18 (16.1)	
	Asian	20 (4.8)	23 (4.9)		1 (1.2)		
	American Indian or Alaska Native	2 (0.5)	3 (0.6)				
	Other	13 (3.1)	15 (3.2)		7 (8.1)	14 (12.5)	
	Pacific Islander	3 (0.7)	3 (0.6)		1 (1.2)	1 (0.9)	
Hispanic or Latino ethnic group, n (%)	Hispanic or Latino	84 (19.5)	101 (20.8)	0.699	15 (17.4)	10 (8.9)	0.116
	Not Hispanic or Latino	346 (80.5)	385 (79.2)		71 (82.6)	102 (91.1)	
MRD 1 Status, n (%)	Positive	158 (40.9)	102 (23.2)	**<0.001**	42 (49.4)	34 (33.0)	**0.033**
	Negative	228 (59.1)	338 (76.8)		43 (50.6)	69 (67.0)	
Leucocyte counts (10^9/L), n (%)	≥30	190 (43.0)	277 (55.0)	**<0.001**	34 (39.1)	53 (47.3)	0.308
	<30	252 (57.0)	227 (45.0)		53 (60.9)	59 (52.7)	
Risk Group, n (%)	High Risk	90 (20.8)	39 (7.8)	**<0.001**	31 (35.2)	20 (17.9)	**<0.001**
	Standard Risk	310 (71.8)	144 (28.8)		47 (53.4)	39 (34.8)	
	Low Risk	32 (7.4)	317 (63.4)		10 (11.4)	53 (47.3)	
Clinical Trial, n (%)	AAML03P1	19 (4.3)	17 (3.4)	**0.017**			
	AAML0531	215 (48.6)	292 (57.9)				
	AAML1031	208 (47.1)	195 (38.7)				
	AML02				71 (80.7)	87 (77.7)	0.732
	AML08				17 (19.3)	25 (22.3)	
FLT3 ITD, n (%)	Yes	88 (20.0)	77 (15.3)	0.074	16 (18.4)	15 (13.5)	0.459
	No	353 (80.0)	426 (84.7)		71 (81.6)	96 (86.5)	

Note: P value is from Chi-squared unless denoted by * for Two Sample T-test.

Supplementary Table 4: 38 CpG AML signature coefficients and genomic loci according to hg38.						
	38 CpG AML sig coefficients	chrm	start	end	GeneNames	CGIPosition
cg17099306	0.074340099	chr13	49549508	49549510	RCBTB1	.
cg14978242	0.070942652	chr5	80205311	80205313	SERINC5	.
cg10089193	0.06945315	chr16	1090938	1090940	C1QTNF8	S_Shore
cg02678414	0.068423605	chr16	2159838	2159840	TRAF7	S_Shelf
cg14882966	0.06007767	chr2	3651762	3651764	.	Island
cg09890699	0.059516957	chr1	54356179	54356181	SSBP3	N_Shore
cg14458815	0.055677433	chr22	23141813	23141815	RSPH14	Island
cg05800336	0.046168229	chr10	131453805	131453807	.	.
cg00151914	0.046074503	chr17	35731530	35731532	TAF15;RASL10B	Island
cg19706516	0.044310524	chr1	151131392	151131394	RP11-68I18.10	Island
cg11817631	0.039837402	chr11	85811565	85811567	SYTL2	S_Shore
cg00532502	0.027136764	chr1	246789970	246789972	LINC01341;KIF28P	S_Shore
cg05348324	0.017770907	chr11	119797455	119797457	.	.
cg04663203	0.013881106	chr17	47851211	47851213	SP6	Island
cg10591771	0.013116146	chr19	3671119	3671121	AC004637.1;PIP5K1C	N_Shore
cg19357999	0.012882775	chr12	121223958	121223960	P2RX4	.
cg16721321	0.011103672	chr4	1208907	1208909	SPON2;CTBP1-AS	N_Shore
cg01543603	0.007178925	chr16	89403132	89403134	RP1-168P16.2;ANKRD11	.
cg04713531	-0.008016879	chr7	158404595	158404597	PTPRN2	N_Shore
cg06748884	-0.010513706	chr16	11138900	11138902	CLEC16A	.
cg08900363	-0.011277984	chr7	597816	597818	PRKAR1B	.
cg03762237	-0.01152593	chr2	98085052	98085054	.	N_Shore
cg00059652	-0.014232316	chr17	69609356	69609358	LINC01483	.
cg09041251	-0.014263132	chr2	10244393	10244395	.	.
cg17632028	-0.016399536	chr4	652752	652754	PDE6B	N_Shore
cg01052291	-0.01666058	chr6	132715433	132715435	VNN1	.
cg08480739	-0.020439876	chr11	67997549	67997551	UNC93B1	Island
cg05480169	-0.020608166	chr10	133253903	133253905	.	.
cg18964582	-0.021157883	chr1	236393881	236393883	EDARADD	N_Shore
cg10280339	-0.030176878	chr15	65519989	65519991	.	S_Shelf
cg07080653	-0.035892949	chr4	53508081	53508083	FIP1L1;LNX1;LNX1-AS1	.
cg04839706	-0.049786958	chr16	46886162	46886164	GPT2	S_Shore
cg14928764	-0.056551664	chr20	23083970	23083972	CD93	.
cg24355048	-0.059277636	chr14	24575821	24575823	CTSG	.
cg02312559	-0.068133496	chr3	134254413	134254415	.	S_Shelf
cg06339275	-0.071282473	chr3	62948996	62948998	LINC00698	.
cg02905663	-0.076509223	chr8	118111467	118111469	EXT1	N_Shore
cg00521620	-0.095595232	chr6	29466638	29466640	UBDP1	.

Supplementary Table 5: Patient characteristics by 38CpG AML signature groups in the discovery and AML02,08 test cohorts.

		38CpG AML signature in discovery cohort, n=946			38CpG AML signature in AML02,08 cohort, n=200		
		High	Low	P-Value	High	Low	P-Value
n		473	473		111	89	
Age (years), mean (SD)		8.8 (6.6)	10.0 (6.0)	**0.003***	7.9 (6.2)	9.9 (5.6)	**0.020***
Age group (years), n (%)	≥10	215 (45.5)	248 (52.4)	0.037	46 (41.8)	49 (55.7)	0.072
	<10	258 (54.5)	225 (47.6)		64 (58.2)	39 (44.3)	
Sex, n (%)	Female	241 (51.0)	227 (48.0)	0.398	50 (45.0)	36 (40.4)	0.611
	Male	232 (49.0)	246 (52.0)		61 (55.0)	53 (59.6)	
Race or ethnic group, n (%)	White	341 (78.0)	356 (80.2)	0.130	79 (72.5)	63 (70.8)	0.854
	Black or African American	59 (13.5)	43 (9.7)		18 (16.5)	14 (15.7)	
	Asian	24 (5.5)	19 (4.3)		1 (0.9)		
	American Indian or Alaska Native	2 (0.5)	3 (0.7)				
	Other	10 (2.3)	18 (4.1)		10 (9.2)	11 (12.4)	
	Pacific Islander	1 (0.2)	5 (1.1)		1 (0.9)	1 (1.1)	
Hispanic or Latino ethnic group, n (%)	Hispanic or Latino	82 (18.0)	103 (22.4)	0.114	14 (12.7)	11 (12.5)	1.000
	Not Hispanic or Latino	374 (82.0)	357 (77.6)		96 (87.3)	77 (87.5)	
MRD 1 Status, n (%)	Positive	158 (38.7)	102 (24.4)	**<0.001**	50 (48.1)	26 (31.0)	**0.026**
	Negative	250 (61.3)	316 (75.6)		54 (51.9)	58 (69.0)	
Leucocyte counts (10^9/L), n (%)	≥30	214 (45.2)	253 (53.5)	0.013	48 (43.6)	39 (43.8)	1.000
	<30	259 (54.8)	220 (46.5)		62 (56.4)	50 (56.2)	
Risk Group, n (%)	High Risk	85 (18.3)	44 (9.4)	**<0.001**	38 (34.2)	13 (14.6)	**<0.001**
	Standard Risk	323 (69.6)	131 (28.0)		59 (53.2)	27 (30.3)	
	Low Risk	56 (12.1)	293 (62.6)		14 (12.6)	49 (55.1)	
Clinical Trial, n (%)	AAML03P1	18 (3.8)	18 (3.8)	0.375			
	AAML0531	264 (55.8)	243 (51.4)				
	AAML1031	191 (40.4)	212 (44.8)				
	AML02				86 (77.5)	72 (80.9)	0.678
	AML08				25 (22.5)	17 (19.1)	
FLT3 ITD, n (%)	Yes	97 (20.6)	68 (14.4)	**0.016**	20 (18.2)	11 (12.5)	0.370
	No	375 (79.4)	404 (85.6)		90 (81.8)	77 (87.5)	

Note: P value is from Chi-squared unless denoted by * for Two Sample T-test.

	Dx from available clinical data	Age (years)	5mC (ALMA Subtype)	Nanopore genomics	Coverage (x)	Mean read quality (Q)	Sample read N50 (bp)	P(Predicted Subtype)	AML Epigenomic Risk	P(Death) at 5y	38 CpG Hazard Score	38 CpG AML signature
**ALMA_1_BM**	Acute Monocytic Leukemia, FAB M5, t(9;11)	0-5	AML with t(8;16); KAT6A::CREBBP/AML with t(v;11q23); KMT2A-r	No KAT6A, KMT2A fusion detected	2.03	23.5	9985	0.603	Low	0.462	-4.176	Low
**ALMA_2_BM**	Acute Myeloid Leukemia with t(8;21) (q22;q22) [AML1/ETO]	5-13	AML with t(8;21); RUNX1::RUNX1T1	8;21 (RUNX1-RUNX1T1) fusion through IGV	6.517	24.3	13773	0.996	Low	0.286	-16.525	Low
**ALMA_3_BM**	Acute Myeloid Leukemia, With Maturation, FAB M2, t(6;9)	13-39	AML with t(6;9); DEK::NUP214	6;9 (NUP214-r) fusion through IGV	1.137	23	9519	0.988	Low	0.434	-3.408	Low
**ALMA_4_PB**	Acute Myelomonocytic Leukemia, FAB M4 - complex karyotype, t(16;21)(p11.2;q22)[3]	0-5	MPAL T-Lymphoblastic/Myeloid	16;21 (FUS-ERG) fusion through IGV	2.118	24	13704	0.935				
**ALMA_5_PB**	Trisomy 21 - MPN (newborn initial diagnosis)	0-5	AML with NUP98-fusion/MDS-related; secondary myeloid	trisomy 21 detected; nup98 fusion not present	11.46	26.1	12581	0.668	High	0.531	-10.657	Low
**ALMA_6_BM**	relapsed MLL AML - 5% blasts, 90% cellularity	39-60	AML with mutated NPM1	KMT2A-KNL1 fusion; benign NPM1 variants	20.864	24.1	12614	0.946	Low	0.424	-3.603	Low
**ALMA_7_BM**	residual AML - 10% blasts KMT2A rearrangement	60+	MDS-related; secondary myeloid/AML with t(v;11q23); KMT2A-r	KMT2A-CREBBP fusion	15.739	25.6	7321	0.787	Low	0.492	-1.39	High
**ALMA_8_BM**	MDS to AML - 40% blasts, RUNX1T1 and 1 copy gains	39-60	MDS-related; secondary myeloid	SF3B1, KRAS, P53 mutations	7.769	26.1	15686	0.94	High	0.621	-3.908	Low
**ALMA_9_BM**	Myelodysplastic syndrome, NOS (C42.1) - Runx1T1 copy gain with trisomy 8. 15-20% blasts; 80% cellularity	60+	MDS-related; secondary myeloid	RUNX1;RUNX1-AS1 mutations	18.117	25.6	13069	0.911	Low	0.271	-6.771	Low
**ALMA_10_BM**	Acute myelomonocytic leukemia NPM1+, 70-80% blasts	60+	AML with mutated NPM1	NPM1 mutation	12.158	25.6	18101	0.885	Low	0.424	-8.46	Low
**ALMA_11_BM**	Relapsed refractory MLL+, FLT3 TKD + monocytic AML	13-39	AML with t(v;11q23); KMT2A-r	KMT2A-SEPTIN9 fusion	17.296	21.9	5953	0.885	High	0.613	-0.475	High
**ALMA_11_PB**	Relapsed refractory MLL+, FLT3 TKD + monocytic AML	13-39	AML with t(v;11q23); KMT2A-r	KMT2A-SEPTIN9 fusion	24.615	22.7	7315	0.986	High	0.754	-2.51	Low
**ALMA_12_BM**	AML progressed from intermediate/high-risk MDS - Day 68 **s/p** Vyxeos	60+	Otherwise-Normal Control	CDKN2A, P53 mutations	11.25	25.7	19128	0.891				
**ALMA_13_BM**	flow suspicious for myeloblasts (35%) but dim CD19	60+	MDS-related; secondary myeloid	RUNX1;RUNX1-AS1 mutations	14.043	25.8	14878	0.811	High	0.652	-8.607	Low
**ALMA_13_PB**	flow suspicious for myeloblasts (35%) but dim CD19	60+	MDS-related; secondary myeloid	RUNX1;RUNX1-AS1 mutations	7.229	26.2	15912	0.888	High	0.711	-9.02	Low
**ALMA_14_PB**	relapsed/refractory AML - no response after multiple therapies (hiDAC, CD123 AML BiTE, DAC-Venetoclax)	39-60	MDS-related; secondary myeloid	DDX10-SKA3 fusion	14.937	26.3	25911	0.916	High	0.525	-4.038	Low
**ALMA_15_PB**	relapsed/refractory AML - **s/p** DAC-Venetoclax	60+	Not confident	U2AF1, KRAS mutation	16.734	26.6	22267	0.45				
**ALMA_16_BM**	AML w/ MDS changes - **s/p** AraC-Dauno	60+	Otherwise-Normal Control	no significant pathogenic mutations or fusions	18.732	26.3	22632	0.995				
**ALMA_16_PB**	AML w/ MDS changes - **s/p** AraC-Dauno	60+	Otherwise-Normal Control	no significant pathogenic mutations or fusions	17.143	25.9	20304	0.94				
**ALMA_17_PB**	Myeloid leukemia associated with Down Syndrome - 60% blasts	0-5	AML with NUP98-fusion	trisomy 21 detected; nup98 fusion not present	19.356	23.5	4262	0.957	High	0.577	-5.327	Low

Note: Matched bone marrow and peripheral blood samples are shaded in grey. Predictions made using ALMA-classifier v0.1.4[19].

Supplementary References

1 Bertrums, E. J. M. *et al.* Comprehensive molecular and clinical characterization of NUP98 fusions in pediatric acute myeloid leukemia. *Haematologica* **108**, 2044-2058 (2023). https://doi.org/10.3324/haematol.2022.281653

2 Aplenc, R. *et al.* Bortezomib with standard chemotherapy for children with acute myeloid leukemia does not improve treatment outcomes: a report from the Children's Oncology Group. *Haematologica* **105**, 1879-1886 (2020). https://doi.org/10.3324/haematol.2019.220962

3 Bolouri, H. *et al.* A B-cell developmental gene regulatory network is activated in infant AML. *PloS one* **16**, e0259197-e0259197 (2021). https://doi.org/10.1371/journal.pone.0259197

4 Gamis, A. S. *et al.* Gemtuzumab ozogamicin in children and adolescents with de novo acute myeloid leukemia improves event-free survival by reducing relapse risk: results from the randomized phase III Children's Oncology Group trial AAML0531. *Journal of clinical oncology : official journal of the American Society of Clinical Oncology* **32**, 3021-3032 (2014). https://doi.org/10.1200/JCO.2014.55.3628

5 Yamato, G. *et al.* Genome-wide DNA methylation analysis in pediatric acute myeloid leukemia. *Blood Advances* **6**, 3207-3219 (2022). https://doi.org/10.1182/bloodadvances.2021005381

6 Tokumasu, M. *et al.* Adverse prognostic impact of KIT mutations in childhood CBF-AML: the results of the Japanese Pediatric Leukemia/Lymphoma Study Group AML-05 trial. *Leukemia* **29**, 2438-2441 (2015). https://doi.org/10.1038/leu.2015.121

7 Bolouri, H. *et al.* The molecular landscape of pediatric acute myeloid leukemia reveals recurrent structural alterations and age-specific mutational interactions. *Nat Med* **24**, 103-112 (2018). https://doi.org/10.1038/nm.4439

8 Cooper, T. M. *et al.* AAML03P1, a pilot study of the safety of gemtuzumab ozogamicin in combination with chemotherapy for newly diagnosed childhood acute myeloid leukemia: a report from the Children's Oncology Group. *Cancer* **118**, 761-769 (2012). https://doi.org/10.1002/cncr.26190

9 Lange, B. J. *et al.* Outcomes in CCG-2961, a children's oncology group phase 3 trial for untreated pediatric acute myeloid leukemia: a report from the children's oncology group. *Blood* **111**, 1044-1053 (2008). https://doi.org/10.1182/blood-2007-04-084293

10 Cancer Genome Atlas Research, N. *et al.* Genomic and epigenomic landscapes of adult de novo acute myeloid leukemia. *The New England journal of medicine* **368**, 2059-2074 (2013). https://doi.org/10.1056/NEJMoa1301689

11 Giacopelli, B. *et al.* DNA methylation epitypes highlight underlying developmental and disease pathways in acute myeloid leukemia. *Genome research* **31**, 747-761 (2021). https://doi.org/10.1101/gr.269233.120

12 Tyner, J. W. *et al.* Functional genomic landscape of acute myeloid leukaemia. *Nature* **562**, 526-531 (2018). https://doi.org/10.1038/s41586-018-0623-z

13 Cabezón, M. *et al.* Different methylation signatures at diagnosis in patients with high-risk myelodysplastic syndromes and secondary acute myeloid leukemia predict azacitidine response and longer survival. *Clinical Epigenetics* **13** (2021). https://doi.org/10.1186/s13148-021-01002-y

14 Nordlund, J. *et al.* Genome-wide signatures of differential DNA methylation in pediatric acute lymphoblastic leukemia. *Genome biology* **14**, r105-r105 (2013). https://doi.org/10.1186/gb-2013-14-9-r105

15 Schmiegelow, K. *et al.* Long-term results of NOPHO ALL-92 and ALL-2000 studies of childhood acute lymphoblastic leukemia. *Leukemia* **24**, 345-354 (2010). https://doi.org/10.1038/leu.2009.251

16 Alexander, T. B. *et al.* The genetic basis and cell of origin of mixed phenotype acute leukaemia. *Nature* **562**, 373-379 (2018). https://doi.org/10.1038/s41586-018-0436-0

17 Touzart, A. *et al.* Epigenetic analysis of patients with T-ALL identifies poor outcomes and a hypomethylating agent-responsive subgroup. *Science translational medicine* **13** (2021). https://doi.org/10.1126/scitranslmed.abc4834

18 Marchi, F. f-marchi/ALMA: ALMA v0.2.0 (v0.2.0). *Zenodo* (2025). https://doi.org/10.5281/zenodo.15653263

19 Marchi, F. f-marchi/ALMA-classifier: ALMA Classifier v0.1.4 (v0.1.4). *Zenodo* (2025). https://doi.org/10.5281/zenodo.15636415
