# Supplementary material for: Epigenomic diagnosis and prognosis of Acute Myeloid Leukemia
Source: Nat Commun. 2025 Jul 29;16:6961. doi: 10.1038/s41467-025-62005-4 (PMC12307604; doi:10.1038/s41467-025-62005-4)
Supplement: Supplementary file 2 — Reporting Summary [file 41467_2025_62005_MOESM2_ESM.pdf]

nature portfolio

Corresponding author(s): Jatinder Lamba

Last updated by author(s): Jul 8, 2025

Reporting Summary

Nature Portfolio wishes to improve the reproducibility of the work that we publish. This form provides structure for consistency and transparency in reporting. For further information on Nature Portfolio policies, see our Editorial Policies and the Editorial Policy Checklist.

Statistics

For all statistical analyses, confirm that the following items are present in the figure legend, table legend, main text, or Methods section.

n/a	Confirmed	
☐	☒	The exact sample size (n) for each experimental group/condition, given as a discrete number and unit of measurement
☐	☒	A statement on whether measurements were taken from distinct samples or whether the same sample was measured repeatedly
☐	☒	The statistical test(s) used AND whether they are one- or two-sided<br>*Only common tests should be described solely by name; describe more complex techniques in the Methods section.*
☐	☒	A description of all covariates tested
☐	☒	A description of any assumptions or corrections, such as tests of normality and adjustment for multiple comparisons
☐	☒	A full description of the statistical parameters including central tendency (e.g. means) or other basic estimates (e.g. regression coefficient) AND variation (e.g. standard deviation) or associated estimates of uncertainty (e.g. confidence intervals)
☐	☒	For null hypothesis testing, the test statistic (e.g. F, t, r) with confidence intervals, effect sizes, degrees of freedom and P value noted<br>*Give P values as exact values whenever suitable.*
☐	☒	For Bayesian analysis, information on the choice of priors and Markov chain Monte Carlo settings
☒	☐	For hierarchical and complex designs, identification of the appropriate level for tests and full reporting of outcomes
☐	☒	Estimates of effect sizes (e.g. Cohen's d, Pearson's r), indicating how they were calculated

Our web collection on statistics for biologists contains articles on many of the points above.

Software and code

Policy information about availability of computer code

Data collection	Source code for how data was collected, as well as for all analyses, figures, and tables are publicly available at the electronic notebook we created for this study: https://f-marchi.github.io/ALMA/ and as source code (https://github.com/f-marchi/ALMA/releases/tag/v0.2.0; DOI:10.5281/zenodo.15653263. Software and hardware information for all analyses are noted at the end of each chapter of the under section “Watermark”.
Data analysis	With the study, we are releasing alma-classifier v0.1.4 (https://github.com/f-marchi/ALMA-classifier; DOI: 10.5281/zenodo.15636415), enabling others to use, reproduce, and build upon the three models described here. At release (v0.1.4), it is structured as an open-source Python package and Docker image that takes as input methylation values from 331556 CpGs (with some room for missing values) and outputs calculated predictions for ALMA Subtype, AML Epigenomic Risk, and 38-CpG AML Signature.

For manuscripts utilizing custom algorithms or software that are central to the research but not yet described in published literature, software must be made available to editors and reviewers. We strongly encourage code deposition in a community repository (e.g. GitHub). See the Nature Portfolio guidelines for submitting code & software for further information.

Data

Policy information about availability of data

All manuscripts must include a data availability statement. This statement should provide the following information, where applicable:

- Accession codes, unique identifiers, or web links for publicly available datasets
- A description of any restrictions on data availability
- For clinical datasets or third party data, please ensure that the statement adheres to our policy

Discovery (training) raw DNA methylation array data analyzed in this study were obtained from Gene Expression Omnibus (GEO) under accession codes GSE190931, GSE124413, GSE133986, GSE159907, GSE152710, GSE49031, GSE147667, as well as from Genomic Data Commons (GDC; https://portal.gdc.cancer.gov/) under categories GDC-TARGET-AML, GDC-TCGA-AML, GDC-TARGET-ALL. Processed, patient-level methylation and clinical data from the discovery/training cohort are available at DOI:10.5281/zenodo.1565326381 through https://github.com/f-marchi/ALMA/releases/tag/v0.2.0 and Source Data, respectively. TThe raw nanopore genome sequencing data generated in this study are not publicly available because participant consent did not explicitly cover deposition in public repositories. However, participants did consent to the use for research purposes and the data are available for bona fide research upon reasonable request to the corresponding author (jatinderklamba@ufl.edu). Access is subject to approval by the relevant institutional ethics board and completion of a Data Use Agreement (DUA) to ensure compliance with ethical and legal obligations. Processed, de-identified methylation and clinical data are available at the same DOI and in Supplementary Table 6, respectively. The remaining data are available within the Article, Supplementary Information or Source Data file.

Human research participants

Policy information about studies involving human research participants and Sex and Gender in Research.

Reporting on sex and gender	In this study, we reported sex as a biological attribute. In our discovery cohort, the diagnostic model was trained on 711 (50.5%) female patients and the prognostic models were trained on 468 (49.5%) female patients. In the nanopore test cohort, 10 patients were male and 7 were female.
Population characteristics	A total of 20 specimens from 17 patients of all ages (0.02-78 years) were considered for analysis for having a diagnosis of AML/MDS with >1x WGS coverage, comprising 12 BM and 8 PB (3 patients had matched PB and BM).
Recruitment	A cohort of patients from adult and pediatric hematology/oncology units from UF Health Shands Hospital who consented to providing samples to research and were suspected for or diagnosed with acute leukemia were considered for the study.
Ethics oversight	This research complies with all relevant ethical regulations. The study protocols were approved by the University of Florida's Institutional Review Board.

Note that full information on the approval of the study protocol must also be provided in the manuscript.

Field-specific reporting

Please select the one below that is the best fit for your research. If you are not sure, read the appropriate sections before making your selection.

[x] Life sciences [ ] Behavioural & social sciences [ ] Ecological, evolutionary & environmental sciences

For a reference copy of the document with all sections, see nature.com/documents/nr-reporting-summary-flat.pdf

Life sciences study design

All studies must disclose on these points even when the disclosure is negative.

Sample size	3845 samples were downloaded from GEO and GDC (3314 samples after QC and exclusions). These are publicly available datasets selected from GEO and GDC in 2023 based on the following 3 criteria: 1. It must stem from a high-quality clinical trial or a large cohort study. 2. It must contain clearly-labeled diagnostic leukemia samples from bone marrow or peripheral blood. 3. It must be available as raw methylation data (.idat files) from methylation arrays (450k or EPIC). Testing was conducted in 200 patient samples collected in the multicenter AML02 and 08 clinical trials. 20 patient samples were collected from the University of Florida Leukemia Bank.
Data exclusions	Preliminary quality control exclusion criteria included: i) 12003 sex-linked and non-CpGs; ii) 47382 CpGs deemed unreliable based on literature benchmarking; iii) 460 samples due to Illumina quality control p-value failure; iv) 61512 CpGs with over 5% missing values; v) 60 non-hematopoietic samples; vi) 11 samples considered outliers according to PCA analysis. Finally, interpolation by batch mean filled the remaining missing values. To adjust for potential confounding variability, batch correction was performed using ComBat. The final dataset of 331556 CpGs and 3314 samples was considered for downstream statistical analyses (Supplementary Fig 11).
Replication	To abide by the criteria recently proposed in the literature of machine learning applications in life sciences and establish the rigor of our bioinformatic pipelines, we made the raw training data, processed training data, model weights, and source code publicly available and open source. All methods and results from this study were written in step-by-step format using Jupyter Book through GitHub-pages. Additionally, testing samples come from independently conducted clinical trials and independently processed pipelines. Specimen-to-result testing was done in-house using nanopore sequencing to validate findings using distinct platform chemistries.

Randomization	NA
Blinding	NA

Reporting for specific materials, systems and methods

We require information from authors about some types of materials, experimental systems and methods used in many studies. Here, indicate whether each material, system or method listed is relevant to your study. If you are not sure if a list item applies to your research, read the appropriate section before selecting a response.

Materials & experimental systems

n/a	Involved in the study
☒	☐ Antibodies
☒	☐ Eukaryotic cell lines
☒	☐ Palaeontology and archaeology
☒	☐ Animals and other organisms
☒	☐ Clinical data
☒	☐ Dual use research of concern

Methods

n/a	Involved in the study
☒	☐ ChIP-seq
☒	☐ Flow cytometry
☒	☐ MRI-based neuroimaging
